# A Mokken scale analysis of the peer physical examination questionnaire

**DOI:** 10.1186/s12998-018-0176-0

**Published:** 2018-03-01

**Authors:** Brett Vaughan, Sandra Grace

**Affiliations:** 1 College of Health & Biomedicine, Victoria University, Melbourne, Australia; 2 Institute of Sport, Exercise and Active Living, Victoria University, Melbourne, Australia; 3 School of Health & Human Sciences, Southern Cross University, Lismore, Australia

**Keywords:** Evaluation, Item response theory, Osteopathy, Osteopathic medicine, Internal structure

## Abstract

**Background:**

Peer physical examination (PPE) is a teaching and learning strategy utilised in most health profession education programs. Perceptions of participating in PPE have been described in the literature, focusing on areas of the body students are willing, or unwilling, to examine. A small number of questionnaires exist to evaluate these perceptions, however none have described the measurement properties that may allow them to be used longitudinally. The present study undertook a Mokken scale analysis of the Peer Physical Examination Questionnaire (PPEQ) to evaluate its dimensionality and structure when used with Australian osteopathy students.

**Methods:**

Students enrolled in Year 1 of the osteopathy programs at Victoria University (Melbourne, Australia) and Southern Cross University (Lismore, Australia) were invited to complete the PPEQ prior to their first practical skills examination class. R, an open-source statistics program, was used to generate the descriptive statistics and perform a Mokken scale analysis. Mokken scale analysis is a non-parametric item response theory approach that is used to cluster items measuring a latent construct.

**Results:**

Initial analysis suggested the PPEQ did not form a single scale. Further analysis identified three subscales: ‘comfort’, ‘concern’, and ‘professionalism and education’. The properties of each subscale suggested they were unidimensional with variable internal structures. The ‘comfort’ subscale was the strongest of the three identified. All subscales demonstrated acceptable reliability estimation statistics (McDonald’s omega > 0.75) supporting the calculation of a sum score for each subscale.

**Conclusion:**

The subscales identified are consistent with the literature. The ‘comfort’ subscale may be useful to longitudinally evaluate student perceptions of PPE. Further research is required to evaluate changes with PPE and the utility of the questionnaire with other health profession education programs.

## Background

Peer physical examination (PPE) is a key pedagogical strategy in health professional education. Its benefits are well documented and include developing respect and sensitivity to patient’s needs [1] and understanding the patient experience ‘from the inside’ as students undress, disclose personal information and act as models for examination by fellow students [2]. PPE also provides the opportunity to develop the level of clinical competence required for practising on real patients [3] and different body types [4].

A number of tools have been developed to assess attitudes to PPE. The most common approach is by survey of students. The ‘Examining Fellow Students’ questionnaire or a variation of it has been frequently used [2, 5–10]. The questionnaire was first developed in 1998 by O’Neill et al. [11] and further developed to a whole of body approach by Chang and Power [12] who also evaluated its face validity and pilot tested it with a convenience sample. Using this whole of body approach both sensitive regions (e.g. groin, rectal and external genitals) and non-sensitive regions (e.g. hands, feet, head) could be included. The survey was also adapted by Rees et al. [13] and used to ask medical students to indicate which of eleven body parts they would not be willing to examine or have examined by a same or opposite gender peer.

Chang and Power [12] developed a 25-item questionnaire to explore students’ perceptions of PPE addressing 3 a-priori domains: (1) comfort with PPE; (2) professionalism, appropriateness, and perceived value of PPE; and (3) willingness to perform peer breast, genital, and rectal examinations. Consorti et al. [8] developed the ‘Peer Physical Examination Questionnaire’ (PPEQ) and used it in conjunction with the ‘Examining Fellow Students’ questionnaire as a point of reference for evaluating criterion validity. Other surveys used to assess students’ attitudes to PPE include one that explored nursing students’ confidence in measuring blood pressure on their peers [14] and others that focused on the pedagogy of PPE, including student perceptions of dyad learning [15], partnering [16], and informed consent [17, 18]. Pols et al. [19] surveyed medical students about the incidence and consequences of medical problems discovered during PPE.

Other researchers surveyed clinical educators. In one study clinical educators were sent a list of 14 common invasive and non-invasive clinical procedures and asked if they allowed students to practise them on each other, and if so, what level of consent was used [20]. Another study sought medical clinical educators’ responses to two questions: (1) did their students participate in PPE, and (2) did they have a PPE policy. Respondents were invited to send a copy of their policy to the researchers for further analysis.

Despite the number of tools for assessing attitudes to PPE and the frequency of their use reported in the literature, evidence for the validity of the scores derived from these questionnaires is limited. Wismeijer et al. [21] suggest that “given the uncertainty about the true dimensionality of the data, a second opinion provided by a conceptually different method may be very useful” (p. 324). The aim of this study was to investigate the dimensionality and structure of one of these evaluation tools – the Consorti et al. [8] 16-item PPEQ.

## Methods

The present study forms part of a larger investigation into PPE perceptions in two Australian osteopathy programs: one at Victoria University (VU) (Melbourne, Australia), and the other at Southern Cross University (SCU) (Lismore, Australia). Human Research Ethics Committees at both institutions approved the study.

### Participants

Students enrolled in Year 1 of the osteopathy programs at VU and SCU were invited to participate in the study as part of their first practical skills class in both the 2015 and 2016 academic years. Students were informed by email and notice on the respective university learning management systems that the study was taking place. The Information to Participants sheets were made available on the learning management systems and in the practical skills classes.

### Measure

Participants completed a demographic questionnaire, the ‘Examining Fellow Students’ questionnaire and the PPEQ. Responses were anonymous, however students self-generated a code in order to match their responses as part of the larger longitudinal study. The PPEQ is the focus of the current evaluation and responses to the ‘Examining Fellow Students’ will be reported elsewhere. The PPEQ utilises a 5-point Likert-type scale (0 – strongly disagree, 4 – strongly agree) to evaluate students’ perceptions of PPE. Principal components analysis of the PPEQ identified three components: ‘appropriateness and usefulness’, ‘sexual implications’ and ‘passive role’ accounting for 62.8% of the variance and a Cronbach’s alpha of 0.86 [8].

### Data analysis

Data were entered into SPSS (IBM Corp, USA) with items 3 to 7 and item 12 recoded as per Consorti et al. [8]. After rescoring, data were exported to *R* [22] for analysis using the *psych* [23] and *mokken* [24] packages. Descriptive data analysis was undertaken using the *psych* package.

#### Mokken scale analysis

The *mokken* package was used to perform a Mokken scale analysis (MSA) following the procedures and steps described by Stochl et al. [25] and Van Der Ark [24]. MSA is a non-parametric item response theory (IRT) approach that is used to evaluate dimensionality (number of concepts measured by the questionnaire) and the internal structure (relationship between the items) of a scale or questionnaire [26, 27]. IRT models attempt to fit the data to ‘S’ or sigmoid-shaped curves as this shape represents the expected responses to an individual questionnaire item. MSA is not as restrictive with respect to fit of data to sigmoid-shaped item response curves, particularly when compared to more restrictive IRT models such as Rasch analysis [25]. This approach (MSA) may lead to an increase in the number of useful items being retained that would otherwise be removed in a more restrictive IRT model. The creation of a Mokken scale is based on the assumptions of unidimensionality (the data measures a single latent construct), monotonicity (the item characteristic curve demonstrates higher values on the curve for higher values of the latent construct), and local independence (the responses to items measuring the latent construct are independent of the response to every other item on the scale) [25]. For ordinal data, the double monotonicity model (DMM) for Mokken scaling is used and adds the additional assumption of non-intersecting item characteristic curves [25]. To meet the requirements of a Mokken scale for this model, the basic assumptions described previously must be met. When these requirements are met, the total score can be said to represent the latent construct or the concept theoretically being measured by the items in the questionnaire. Further, the DMM model may also allow for the identification of invariant item ordering, that is, the ordering of the items on the latent construct continuum does not change regardless of the level of the latent construct demonstrated by the respondent.

The following describes how the MSA was performed in the current study in *R*. The first step in MSA is to evaluate the items that may form Mokken scales using the automated item search function (*aisp*) [28]. All 16 PPEQ items were evaluated to identify potential Mokken scales using the initial cut off of 0.3, then increasing incrementally in 0.05 steps until the scales could no longer be logically explained and the analysis resulted in two or more scales [28], as would be consistent with the PPE literature. Once a scale was identified, the scalability coefficients for all items creating a scale (H), the individual items in that scale (H*i*), and the item pairs (H*ij*) were calculated along with the standard error [29]. A scale is said to be ‘weak’ if H is less than 0.3, ‘moderate’ if H is between 0.4–0.5, and ‘strong’ if H is greater than 0.5. Items are thought to be suitable for inclusion in a Mokken scale if they demonstrate a H*i* value greater than 0.3, and the H*ij* value is greater than 0. Next, local dependence was checked using the conditional association procedure [30]. Where an item pair was identified as locally dependent, the item with the lower H*i* value was removed and the data set reanalysed. Monotonicity was then checked using both graphical and numerical approaches to ensure that each item demonstrated a monotonically increasing item response function. Invariant item ordering (H^T^) was then evaluated with values less than 0.3 suggesting the items could not be meaningfully ordered, with items of between 0.4–0.5 demonstrating moderate ordering, and with items with H^T^ greater than 0.5 demonstrating strong item ordering. Once a scale had been finalised, Mokken’s *rho* was evaluated as one of the reliability estimations with a value over 0.7 being acceptable [31].

#### Reliability estimation

In addition to Mokken’s *rho,* McDonald’s omega (ω) [32–34] was the selected reliability estimation method and calculated using the *psych* package. Green and Yang [35] have suggested that the use of Cronbach’s alpha is limited given the propensity for data in the educational and psychological sciences to violate the assumptions underlying its proper use. The *psych* package [23] in *R* [22] presents *ω* as hierarchical ω_h_ and total ω_t_. ω_t_ provides an indication as to the reliability of the general factor, and ω_h_ is the proportion of the total score variance that can only be attributed to the general factor [36]. Zinbarg et al. [33] suggest that ω_h_ is the most accurate reliability estimate in most situations. High ω_h_ values suggest that the general factor accounts for the total score variance, and supports unidimensionality [37]. ω_h_ values greater than 0.7 support calculation of the total score [36]. Both McDonald’s omega total (ωt) and omega hierarchal (ωh) were calculated: ωt is the reliability of the total score; and, ωh is the proportion of variance due to the latent construct and provides an indication about the extent to which the total questionnaire score estimates the latent construct [32] – perception of PPE.

## Results

Three hundred and fourteen students (*N* = 314) completed the PPEQ at the start of the first teaching period in 2015 (*n* = 153) and 2016 (*n* = 161) representing an overall 90% response rate. Two hundred and thirty responses were from VU (*n* = 230, 73.2%). Descriptive statistics for the PPEQ after rescoring are presented in Table 1.

**Table 1:** Descriptive statistics and initial scalability (*Hi* and standard error) for the Peer Physical Examination Questionnaire (PPEQ) items

PPEQ item	Mean (SD)	Median	H*i* (standard error)
1. In general, I feel comfortable when performing PPE on a colleague of mine	3.24 (0.72)	3	0.47 (0.03)
2. In general, I feel comfortable when a colleague performs PPE on me	3.18 (0.75)	3	0.47 (0.03)
3. I feel embarrassed if I am undressed for PPE in front of my group of colleagues^a^	2.47 (1.11)	2	0.44 (0.03)
4. I feel embarrassed if I am undressed for PPE in front of my teacher or tutor^a^	2.60 (1.10)	3	0.42 (0.03)
5. I am concerned of being a possible object of sexual interest during PPE^a^	3.33 (0.82)	4	0.26 (0.03)
6. I am concerned of experiencing possible sexual interest for my colleagues during PPE^a^	3.39 (0.83)	4	0.27 (0.04)
7. I am concerned of experiencing possible sexual interest for my teacher or tutor during PPE^a^	3.61 (0.72)	4	0.27 (0.05)
8. I feel comfortable when performing PPE on a colleague of my same sex	3.44 (0.67)	4	0.45 (0.04)
9. I feel comfortable when performing PPE on a colleague of the opposite sex than mine	3.21 (0.80)	3	0.48 (0.04)
10. I feel comfortable when PPE is performed on me by a colleague of my same sex	3.44 (0.63)	4	0.47 (0.03)
11. I feel comfortable when PPE is performed on me by a colleague of the opposite sex than mine	3.22 (0.76)	3	0.50 (0.03)
12. It is inappropriate to perform PPE on persons that will be my future colleagues^a^	3.37 (0.93)	4	0.24 (0.05)
13. To perform PPE is an appropriate practice for the education of a medical doctor (osteopath)	3.69 (0.67)	4	0.34 (0.06)
14. To undergo PPE is an appropriate practice for the education of a medical doctor (osteopath)	3.73 (0.56)	4	0.40 (0.04)
15. In performing PPE I get useful feedback from my colleagues about my skill	3.70 (0.50)	4	0.44 (0.04)
16. It is a sign of professionalism as a student to accept to perform and undergo PPE	3.57 (0.66)	4	0.35 (0.04)

^a^items were rescored as per Consorti et al. [8]

### Mokken scale analysis

Prior to the analysis responses from eight students were removed due to missing data. Scalability of the full 16-item PPEQ is presented in Table 1. Individual item scalability (*Hi*) for items 5, 6, 7, and 12 were below the accepted cut-off of 0.30 [25], with item 13 falling below this cut-off if the standard error is taken into account. The *H* coefficient of 0.40 (0.03) suggests a ‘weak’ scale and is likely multidimensional.

The *aisp* function in the *mokken* package [24] was used to identify potential Mokken or unidimensional scales. As per Stochl et al. [25] the initial lower bound was set at 0.30 (*minvi* size) and subsequent analyses undertaken in increasing 0.05 steps (Table 2).

**Table 2:** Identification of Mokken scales for the Peer Physical Examination Questionnaire (PPEQ) using the *aisp* function

	*minvi* size				
PPEQ item	0.30	0.35	0.40	0.45	0.5
1. In general, I feel comfortable when performing PPE on a colleague of mine	1	1	1	1	1
2. In general, I feel comfortable when a colleague performs PPE on me	1	1	1	1	1
3. I feel embarrassed if I am undressed for PPE in front of my group of colleagues	1	1	1	1	1
4. I feel embarrassed if I am undressed for PPE in front of my teacher or tutor	1	1	1	1	1
5. I am concerned of being a possible object of sexual interest during PPE	1	2	2	2	2
6. I am concerned of experiencing possible sexual interest for my colleagues during PPE	2	2	2	2	2
7. I am concerned of experiencing possible sexual interest for my teacher or tutor during PPE	2	2	2	2	2
8. I feel comfortable when performing PPE on a colleague of my same sex	1	1	1	1	1
9. I feel comfortable when performing PPE on a colleague of the opposite sex than mine	1	1	1	1	1
10. I feel comfortable when PPE is performed on me by a colleague of my same sex	1	1	1	1	1
11. I feel comfortable when PPE is performed on me by a colleague of the opposite sex than mine	1	1	1	1	1
12. It is inappropriate to perform PPE on persons that will be my future colleagues	x	x	x	x	x
13. To perform PPE is an appropriate practice for the education of a medical doctor (osteopath)	1	1	3	3	3
14. To undergo PPE is an appropriate practice for the education of a medical doctor (osteopath)	1	1	1	3	3
15. In performing PPE I get useful feedback from my colleagues about my skill	1	1	1	3	3
16. It is a sign of professionalism as a student to accept to perform and undergo PPE	1	1	3	3	3

1 = scale one, 2 = scale two, 3 = scale three

The lower bound values suggested a three-scale structure was most appropriate and that item 12 should be removed. The three subscales were identified as ‘comfort’, ‘concern’, and ‘professionalism and education’. Each scale was evaluated for unidimensionality, monotonicity, and invariant item ordering (IIO) as per Van der Ark [24] and Stochl et al. [25].

### Comfort subscale

The ‘comfort’ subscale consisted of PPEQ items 1–4 and 8–11. The *H*-coefficient was 0.61 (±0.03) and *Hi* coefficients were greater than 0.50 and all *Hij* coefficients were non-negative. One non-significant monotonicity violation was identified for each of items 3 and 4. Non-significant IIO was identified for items 1, 2, 9 and 11 however backward selection did not suggest removing any item. *H*^*T*^ was 0.61 indicating high accuracy of the item ordering [38]. These results provide evidence for the unidimensionality of the ‘comfort’ subscale that meets the requirements of a Mokken scale.

### Concern subscale

The ‘concern’ subscale consisted of PPEQ items 5 to 7. The *H*-coefficient was 0.71 (±0.05), all *Hi* coefficients were greater than 0.60 and all *Hij* coefficients were non-negative. No violations of monotonicity were identified. Non-significant IIO was identified for items 5 and 6, and backward selection did not suggest removing either item. *H*^*T*^ was 0.35 indicating low accuracy of the item ordering [38]. These results suggest the ‘concern’ subscale is unidimensional and meets the requirements of a Mokken scale, although the ability to discern between levels of student concern with participating in PPE is limited.

### Professionalism and education subscale

PPEQ items 13 to 16 comprise the ‘professionalism and education’ subscale. The *H*-coefficient was 0.71 (0.05), all *Hi* coefficients were greater than 0.60 and all *Hij* coefficients were non-negative. No violations of monotonicity or IIO were identified. H^T^ was 0.17 indicating limited accuracy of the item ordering. These results suggest the ‘professionalism and education’ subscale is unidimensional and meets the requirements of a Mokken scale, although the ability to discern between different levels of perceived ‘professionalism and education’ associated with PPE is negligible.

### Reliability estimation

McDonald’s omega was calculated as the reliability estimate and the results are presented in Table 3. All values for both ωt and ωh where above an acceptable level. ωh values obtained in the present study provide support for the calculation of a total score for each subscale [36] given that over three-quarters of the variance in the summed score for each subscale is attributable to the latent constructs of ‘comfort’, ‘concern’, and ‘professionalism and education’ respectively.

**Table 3:** Reliability estimates for the three Peer Physical Examination Questionnaire (PPEQ) subscales using McDonald’s omega (ω)

Scale	Omega total (ωt)	Omega hierarchal (ωh)
Comfort	0.95	0.85
Concern	0.90	0.84
Professionalism & education	0.89	0.75

## Discussion

The present study sought to evaluate the dimensionality and structure of the PPEQ developed by Consorti et al. [8]. Research by the current authors has previously identified the need to explore the properties of the questionnaire to provide evidence for its ongoing use as a PPE evaluation tool [39]. Consorti et al. [8] calculated a total score for the PPEQ and used this score as part of their analyses in Italian medical and osteopathy students. However, Mokken scale analysis of the full 16-item PPEQ suggested that the calculation of a total score for the PPEQ is not valid in an Australian osteopathy student population.

Consorti et al. [8] used a Principal Components Analysis (PCA) to evaluate the internal structure of the PPEQ. Wismeijer et al. [21] suggest the use of a number of analytic approaches, including Mokken scale analysis, as a complementary data analysis approach to evaluate dimensionality and different levels of an underlying latent construct given this information is not obtainable from a PCA. Consorti et al. [8] identified a 3-component structure for the PPE in their study (‘appropriateness and usefulness’, ‘sexual implications’ and ‘passive role’) however the same structure was not identified in the present study. Through the Mokken scale analysis, three Mokken scales were identified in the present study: ‘comfort’, ‘concern’, and ‘professionalism and education’. The present study provides evidence for an alternative psychometric structure for the PPEQ that comprises three unidimensional subscales where the summed score for each subscale represents the respective latent construct. This alternative structure has similarities with the *a-priori * domains of ‘comfort with PPE’, and ‘professionalism, appropriateness, and perceived value’ of PPE as described by Chang and Power [12]. Given the similarities, it may be that these are two key themes in the evaluation of PPE.

To create the three PPEQ subscales, it is suggested that item 12 be removed as it did not fit into any of the three Mokken scales identified in the analysis. This item, ‘It is inappropriate to perform PPE on persons that will be my future colleagues’*,* does not appear to measure a construct consistent with the other PPEQ items. The use of the term ‘inappropriate’ may account for this as it is the only item with this term. More frequently used terms in the PPEQ are ‘comfortable’, ‘concerned’ and ‘embarrassed’. Further, students in the present study may ascribe a different meaning to the term ‘inappropriate’. Students in the present study completed the PPEQ on their first day before they had participated in a practical skills class. Their frame of reference for what constitutes ‘inappropriate’ is likely to be different from other students. Another possibility is the translation of the term to ‘inappropriate’ from the initial validation study with Italian osteopathy and medical students [8]. While the item may have had a particular meaning in the initial study, its meaning within the context of the item has been lost.

### ‘Comfort’ subscale

‘Comfort’ with PPE needs to be evaluated as students are often expected to participate in such activities during their pre-professional program [7, 40]. This subscale consisted of items 1–4 and 8–11. Items 1 to 4 gauge the students’ perception with regard to performance of PPE and exposure of their body. Students appear to be generally comfortable with participating in PPE [12, 39]. Items 8 to 11 specifically address comfort with PPE based on sex. The literature suggests that gender has a significant influence on student perceptions of PPE. Discomfort with examining students of a different gender has been identified by females due to fear of sexual exploitation, but also by males for fear of accusations of harassment [40]. Students’ perceptions of these issues are likely to be captured in this PPEQ subscale. Given the increasing awareness of gender diversity it may be that these concerns are not limited to different gender interactions. This has yet to be considered in the literature and provides an avenue for further research. The ‘comfort’ subscale is the strongest of the three PPEQ subscales from a scalability and item ordering standpoint. This suggests that the *comfort* subscale could potentially measure changes in a students’ perception of comfort with PPE over a period of time.

### ‘Concern’ subscale

Much of the literature on PPE relates to *concern* about participating in such activities. The focus of the PPEQ items in this subscale relates specifically to ‘sexual interest’ from not only other students but also the academic and clinical teaching staff. Female students have been reported to be more likely than males to fear critical and teasing comments, and sexual objectification [41]. As highlighted in the discussion of the ‘comfort’ subscale, it may be that this unease extends beyond the reported female/male interaction, however such an assertion has not been described in the literature. Concern has also been expressed about the “immaturity” of fellow students and about potential sexual harassment [42]. Wearn et al. [2] also suggest that issues may arise where students may be (or have previously been) close friends, housemates, or a sexual partner of their peer examiner/examinee, and therefore have blurred boundaries within the context of PPE. From a psychometric standpoint, the item ordering value (*H*^*T*^) was low suggesting that the ability to discern between different levels of perceived concern associated with PPE is negligible albeit a total score can be calculated for this subscale.

### ‘Professionalism and education’ subscale

*‘*Professionalism and education’ are key components in the evaluation of PPE, a position supported by the inclusion of this domain in the study by Chang & Power [12]. Setting professional behaviour standards [12], undertaking a formal PPE participation consent process [18] and creating a positive education environment may contribute to a positive perception of PPE [39]. This subscale also captures the students perceptions about the need to participate in PPE, a theme consistent with other work [6]. As with the ‘concern’ subscale, the item ordering value was low suggesting that the ability to discern between different levels of perceived professionalism and education associated with PPE is negligible. This assertion is potentially supported by Vaughan & Grace [39] who reported no difference in perception of first year osteopathy students in the 4 items of the PPEQ making up this scale over a 12-week period. Evidence is provided for the calculation of a sum score for the subscale.

### Reliability estimations

All three PPEQ subscales demonstrated high scalability coefficients suggesting they are unidimensional and the present study provides support for the their measurement of the underlying latent constructs, namely, ‘comfort’, ‘concern’, and ‘professionalism and education’ associated with PPE. For all three subscales the ωt values were approximately 0.90 suggesting that 90% of the variance in the total score for each subscale is accounted for by the respective latent construct. Calculation of a total score for each subscale (after rescoring where required) is supported by the high ωh values [36]. This subscale score calculation is at odds with Consorti et al. [8] who calculated a total score for the PPEQ and such a position is not supported by the data in the current study.

### Study limitations

There are a number of limitations in the present study. First, the study only explored the opinions of students in two Australian osteopathy programs. Therefore the generalisability of these results to other osteopathy programs, and other health professions is limited. Another limitation is that the study did not evaluate whether the structure of the questionnaire was different with different cultural groups. Acceptance and potentially participation in PPE is known to vary with different cultures [40] and this could result in a different questionnaire structure. It is also possible that a degree of bias was introduced with approximately three-quarters of the data in the present study obtained from one institution.

### Future research and questionnaire use

The PPEQ as described in the current study has a number of uses both in the classroom and research settings. The questionnaire has the potential to be used as an evaluation of the learning environment to identify systemic concerns with participating in PPE beyond the individual student level. Systemic concerns could be addressed using Grace et al.’s [43] strategies for improving the experience of PPE, including the use of written consent forms and formal feedback. The PPEQ can also be used as part of a formal feedback strategy that could be used to inform educators about changes that can be made to improve or modify the PPE experience. The PPEQ also has the potential to be used in longitudinal evaluations of PPE experiences to evaluate changes in perception over time, particularly where students are participating in PPE on a regular basis. Grace et al. [43] suggest that “…providing students with written information about what to expect, and about the pedagogical benefits of experiential learning [PPE], and discussing ethical issues that could be associated with experiential learning could be readily implemented in practical classes” (p. 29). The PPEQ could be used to evaluate perceptions about PPE before and after the provision of this information.

The influence of gender diversity and sexuality as factors influencing PPE participation is an avenue for further research given the literature has thus far only considered female/male interactions. Further work could also explore whether the PPEQ measurement properties are retained in different cultural and gender diverse populations.

## Conclusion

This study provides evidence for the dimensionality and structure of a 15-item version of the ‘Peer Physical Examination Questionnaire’. The current research has identified three subscales within the PPEQ (‘comfort’, ‘concern’, and ‘professionalism and education’) that can be used to explore students’ perceptions of PPE. Calculation of a total score for the modified 15-item scale is not supported, however it is possible to calculate a sum score for each of the three subscales. These subscales have the potential to provide an avenue for further research, including longitudinal changes in perception, particularly as the subscale themes are consistent with the literature. The current research has strengthened the psychometric properties of the PPEQ and others are encouraged to explore the use of the modified questionnaire in their student cohorts.
